# Demographic and regional trends in pneumonia and sepsis related mortality in the U.S., 1999–2020: a CDC wonder database analysis

**DOI:** 10.3389/fpubh.2026.1715043

**Published:** 2026-05-13

**Authors:** Taha Alam, Dhvanit Rajdeep, Sohaima Kamal, Sadaf Siddiqui, Syed Ali Waheed, Muhammad Shuraim Chola, Muhammad Shaheer Bin Faheem, Mohammed Hammad Jaber Amin

**Affiliations:** 1 Dow University of Health Sciences, Karachi, Pakistan; 2 Caucasus International University, Tbilisi, Georgia; 3 Wah Medical College, Wah, Pakistan; 4 Karachi Institute of Medical Sciences, KIMS, Karachi, Pakistan; 5 Alzaiem Alazhari University, Khartoum, Sudan

**Keywords:** CDC WONDER, demographics, mortality trends, pneumonia, sepsis

## Abstract

**Background:**

Pneumonia and sepsis remain major health issues in the United States. Despite a high mortality burden and interconnected nature, they remain underexplored in terms of population-level mortality trend analyses. Understanding their impact is essential for improving therapeutic strategies and reducing preventable deaths.

**Objective:**

We aimed to explore long-term demographic and geographical trends of pneumonia and sepsis-related mortality in the United States from 1999 to 2020.

**Methods:**

The CDC WONDER national database was utilized to analyze differences in mortality associated with pneumonia and sepsis. Age-adjusted mortality rates (AAMRs) per 100,000 persons were calculated, and Jointpoint Regression Program was used to determine the trends in mortality.

**Results:**

The results demonstrated that 806,434 deaths occurred during the study period, with a significant increase in AAMR from 16.66 in 1999 to 25.06 in 2020. Males consistently maintained higher AAMRs than females throughout the observed period (overall AAMR men: 21.16 vs. women: 13.94). AAMRs were highest among NH African Americans and lowest among NH Asians or Pacific Islanders. AAMR also varied substantially by region (overall AAMR: Midwest 14.45; South: 18.82; West: 16.92; Northeast: 16.21). Similarly, higher AAMRs were observed in non-metropolitan areas (17.32) compared with metropolitan areas (16.86).

**Conclusion:**

Despite all the limitations, the findings provide a valuable foundation for future research and targeted public health interventions.

## 1 Introduction

Pneumonia and sepsis are categorized amongst the most significant causes of morbidity and mortality across the globe. Pneumonia, particularly Community Acquired Pneumonia (CAP), alone caused 2.1 million deaths in 2021 (1), affecting especially the older adults, immunocompromised individuals, and children under five (2, 3). In the United States, pneumonia remains a leading cause of hospitalization among adults, contributing to nearly 41,000 deaths each year (4). It ranks among the top 10 causes of mortality, placing a huge burden on the healthcare system (5). Sepsis, which is often precipitated by pneumonia, was responsible for approximately 11 million deaths in 2017, representing 19.7% of all global deaths (6). These trends show the significant and shared global burden of both conditions.

Despite these alarming numbers, the treatment of pneumonia and sepsis remains challenging due to their complex clinical features and high-risk populations. Even with the advancements in antimicrobial therapy, vaccination programs, and supportive care, pneumonia proves to be fatal, thus adding significantly to the global burden of mortality (7). At the same time, sepsis continues to be a leading cause of mortality in intensive care units globally, often arising as a complication of severe infections such as pneumonia (6, 8). Together, they represent a continuum of disease where timely recognition and intervention are critical to improving outcomes.

The implementation of early recognition protocols, rapid initiation of antibiotics, and advances in supportive care have displayed a modest decline in short-term sepsis mortality (9, 10). However, long-term mortality and morbidity among sepsis survivors remain high, with many individuals experiencing frequent hospitalizations, persistent organ dysfunction, and poor quality of life (11, 12). In addition to that, the introduction of pneumococcal vaccination has contributed to a significant decrease in deaths related to pneumonia. However, the overall mortality remains substantial, particularly among aging populations and those with chronic comorbid conditions (13, 14).

According to recent studies, sepsis accounts for nearly one in five deaths worldwide, highlighting the need for reestablishment of prevention and management strategies (6). It has also been shown that pneumonia is the most frequently identified cause of infection-associated sepsis, highlighting the close interrelationship between the two conditions. Therefore, it is important to analyze the mortality trends of both conditions concurrently (15, 16). The recently discovered alterations in pathogen distribution, increasing antimicrobial resistance, and evolving demographic patterns are deemed to strongly influence future mortality trends. Thus, necessitating the performance of ongoing surveillance and adaptation of treatment strategies (17, 18).

Despite a high mortality burden and interconnected nature, pneumonia and sepsis have received limited attention in terms of population-level analysis of mortality trends across nations. To address this gap, we analyzed mortality data from the Centers for Disease Control and Prevention (CDC) WONDER database between 1999 and 2020, focusing on pneumonia and sepsis across United States regional disparities and the evolving burden of pneumonia and sepsis over two decades.

## 2 Methods

### 2.1 Study settings and population

In this retrospective population-based study, we retrieved death certificate data from the CDC WONDER (Centers for Disease Control and Prevention Wide-Ranging Online Data for Epidemiologic Research) database. We analyzed information from death certificates related to pneumonia and sepsis-associated mortality, including adults aged 25 and above in the US from 1999 to 2020. The study included data from all 50 states and the District of Columbia. Mortality statistics were acquired from the Multiple Cause-of-Death Public Use records. Deaths were identified using the following International Statistical Classification of Diseases and Related Health Problems-10th Revision (ICD-10) codes: J12-J18, and A02.1, A20.7, A22.7, A26.7, A32.7, A40.0, A40.1, A40.2, A40.3, A40.8, A40.9, A41.0, A41.1, A41.2, A41.3, A41.4, A41.5, A41.8, A41.9, A42.7, and B37.7. These codes have also been used in prior studies (19, 20). For the purpose of this analysis, deaths were included only when both pneumonia and sepsis were listed anywhere on the death certificate, either as the underlying cause of death or as contributing causes. Deaths in which only pneumonia or only sepsis was listed were not included in the primary analytic cohort. Thus, the study reflects mortality characterized by the co-occurrence of both conditions rather than either condition independently. We ensured transparency in reporting by adhering to Strengthening the Reporting of Observational Studies in Epidemiology (STROBE) guidelines for observational research. Additionally, the use of publicly available data exempts the study from institutional review board (IRB) regulation (21).

### 2.2 Data extraction

Data was extracted and verified for pneumonia- and sepsis-related deaths, encompassing various demographics, population size, location of death, states, regions, years, and metropolitan and non-metropolitan classifications. The demographic of the population included sex, age, race/ethnicity, and location of death, including medical facilities (outpatient, emergency room, inpatient, death on arrival, or status unknown), home, hospice, and nursing home/long-term care facility. We categorized data on the basis of the following race/ethnicity groups: Hispanic or Latino, non-Hispanic (NH) White, NH Black or African American, NH American Indian or Alaska Native, and NH Asian or Pacific Islander, as utilized in previous CDC WONDER database analyses. For classification on the basis of urbanization, we used the National Center for Health Statistics Urban-Rural Classification Scheme, consolidating six categories into two: metropolitan (including medium metro, small metro, large fringe metro, and large central metro areas) and non-metro (consisting of non-core rural areas and micropolitan areas) (22). Age categories were grouped into 25–44 years, 45–64 years, and 65 years and older. Census regions were categorized into the Midwest, West, East, and South as per the CDC WONDER Census Bureau Classifications.

### 2.3 Statistical analysis

To assess national trends in pneumonia- and sepsis-related mortality, we calculated crude mortality rates (CMRs) and age-adjusted mortality rates (AAMRs) per 100,000 population from 1999 to 2020, stratifying the data by age, sex, ethnicity/race, state, and urban-rural status, including 95% confidence intervals (CIs). Crude mortality rates were calculated by dividing the number of pneumonia and sepsis-related deaths by the corresponding U.S. population of that year. Age adjustment was conducted using the 2,000 US standard population (23). To analyze long-term mortality trends, Joinpoint Regression Analysis (Joinpoint Version 5.4.0.0, developed by the National Cancer Institute) was utilized to determine the annual percent change (APC) and its corresponding 95% confidence interval in AAMR (24). A significant increase or decrease in APCs was determined if the slope of the mortality trend was significantly different from zero, with statistical significance set at *p* < 0.05 based on 2-tailed *t*-tests.

## 3 Results

From 1999 to 2020, 806,434 deaths were associated with both pneumonia and sepsis among U.S. individuals ≥25 years or older. (Supplementary Table 1). When classified by place of death, the majority of deaths occurred in medical facilities (88.38%), followed by nursing homes/long-term care facilities (5.99%), hospice facilities (2.56%), the decedent's home (1.98%), and other locations (0.80%) (Supplementary Table 2).

### 3.1 Annual trends for pneumonia and sepsis-related AAMR

**Figure 1:**
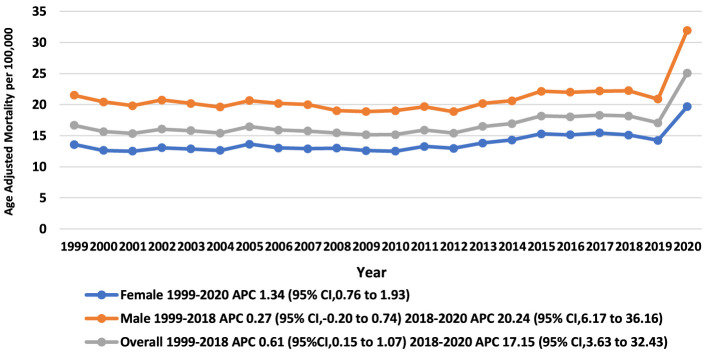
Overall, trends in pneumonia and sepsis-related age-adjusted mortality rates per 100,000 among adults (Aged ≥25) in the United States, 1999 to 2020.

The AAMR for pneumonia and sepsis-related deaths in adults (aged ≥25 years) was 16.66 (95% CI: 16.47 to 16.85) in 1999 and 25.06 (95% CI: 24.86 to 25.25) in 2020. The overall AAMR for deaths related to the concurrence of pneumonia and sepsis showed a notable increase from 1999 to 2018 with an APC of 0.61 (95% CI: 0.15 to 1.07) (*p-*value: 0.011), followed by a significant surge from 2018 to 2020 with an APC of 17.15 (95% CI: 3.63 to 32.42) (*p-*value: 0.014) (Figure 1, Supplementary Tables 3, 4).

### 3.2 Pneumonia and sepsis-related AAMR stratified by sex

During the course of the study, AAMR for men remained consistently higher than that for women (overall AAMR for men: 21.16; 95% CI: 21.1 to 21.23 vs. women: 13.94; 95% CI: 13.9 to 13.98) (Supplementary Figure 2). The AAMR for men in 1999 was 21.5 (95% CI: 21.14 to 21.85), trends for men remained statistically stable (non-significant change) from 1999 to 2018 with an APC of 0.27 (95% CI: 0.20 to 0.74) (*p-*value: 0.246). This was then followed by a sudden surge from 2018 to 2020 with an APC of 20.24 (95% CI: 6.17 to 36.17) (*p-*value: 0.006). While AAMR for women was 13.57 (95% CI: 13.35 to 13.79) in 1999, it notably increased from 1999 to 2020 with an APC of 1.34 (95% CI: 0.76 to 1.93) (*p-*value: 0.0001). The final AAMR for men and women in 2020 was 31.91 (95% CI: 31.58 to 32.24) and 19.67 (95% CI: 19.44 to 19.9), respectively. Although females showed a consistent increasing trend throughout the observed period, male mortality rates did not significantly rise until the 2018–2020 period; however, males maintained higher absolute rates than females throughout (Figure 1, Supplementary Tables 3, 4).

### 3.3 Pneumonia and sepsis-related AAMR stratified by race/ethnicity

**Figure 2:**
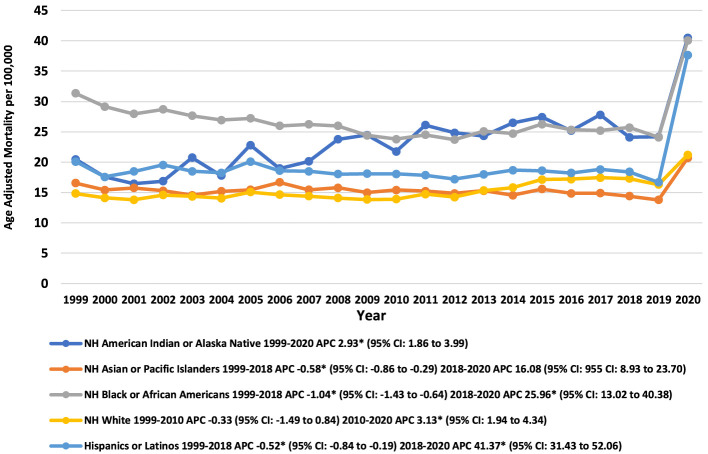
Trends in pneumonia and sepsis-related age-adjusted mortality rates per 100,000, stratified by race and ethnicity among adults (Aged ≥25) in the United States, 1999 to 2020.

From 1999 to 2020, pneumonia and sepsis-related mortality trends varied significantly across the racial groups. The NH African American group experienced the highest overall AAMR, followed by NH American Indians or Alaska Natives, Hispanics or Latinos, NH White, and NH Asian or Pacific Islanders (Figure 2).

Briefly, AAMRs for NH African American, NH Asian or Pacific Islanders, and Hispanics or Latinos declined from 1999 to 2018 with an APC of −1.04 (95% CI: −1.43 to −0.64) (*p-*value: 0.00003), −0.57 (95% CI: −0.85 to −0.29) (*p-*value: 0.0004), and −0.52 (95% CI: −0.84 to −0.19) (*p-*value: 0.004), respectively, while the AAMR for NH White increased from 2010 to 2020 with an APC of 3.13 (95% CI: 1.94 to 4.34) (*p-*value: 0.00003). Following this, the AAMRs for all the mentioned races significantly increased till 2020. The AAMR for NH American Indian or Alaska Native continued to rise throughout the study period with an APC of 2.93 (95% CI: 1.86 to 3.99) (*p-*value: 0.00001) (Supplementary Tables 3, 5).

### 3.4 Pneumonia and sepsis-related AAMR stratified by age group

Pneumonia and sepsis-related mortality rates varied significantly across age groups, with older adults experiencing the highest crude death rates per 100,000 population with an overall AAMR of 68.08 (95% CI: 67.91 to 68.25) (Supplementary Figure 3). Among 25–44-year-old adults, the AAMRs remained steady from 1999 to 2018 with an APC of 0.95 (95% CI: 0.15 to 1.77) (*p-*value: 0.023), which then increased from 2018 to 2020 with an APC of 27.40 (95% CI: 1.64 to 59.70) (*p-*value: 0.037). The AAMR values for 45–64-year-old adults inclined steadily till 2018 with an APC of 1.96 (95% CI: 1.58 to 2.33) (*p-*value < 0.0001) and culminated in a sudden surge from 2018 to 2020 with an APC of 24.67 (95% CI: 13.16 to 37.34) (*p-*value: 0.0001). Older adults (65–85 + years) experienced a significant surge from 2012 to 2020 with an APC of 3.66 (95% CI: 1.59 to 5.76) (*p-*value: 0.001). Overall, the mortality rates followed an expected pattern, with older age groups exhibiting sustained high mortality rates (Supplementary Tables 3, 9).

### 3.5 Pneumonia and sepsis-related AAMR stratified by geographic region

**Figure 3:**
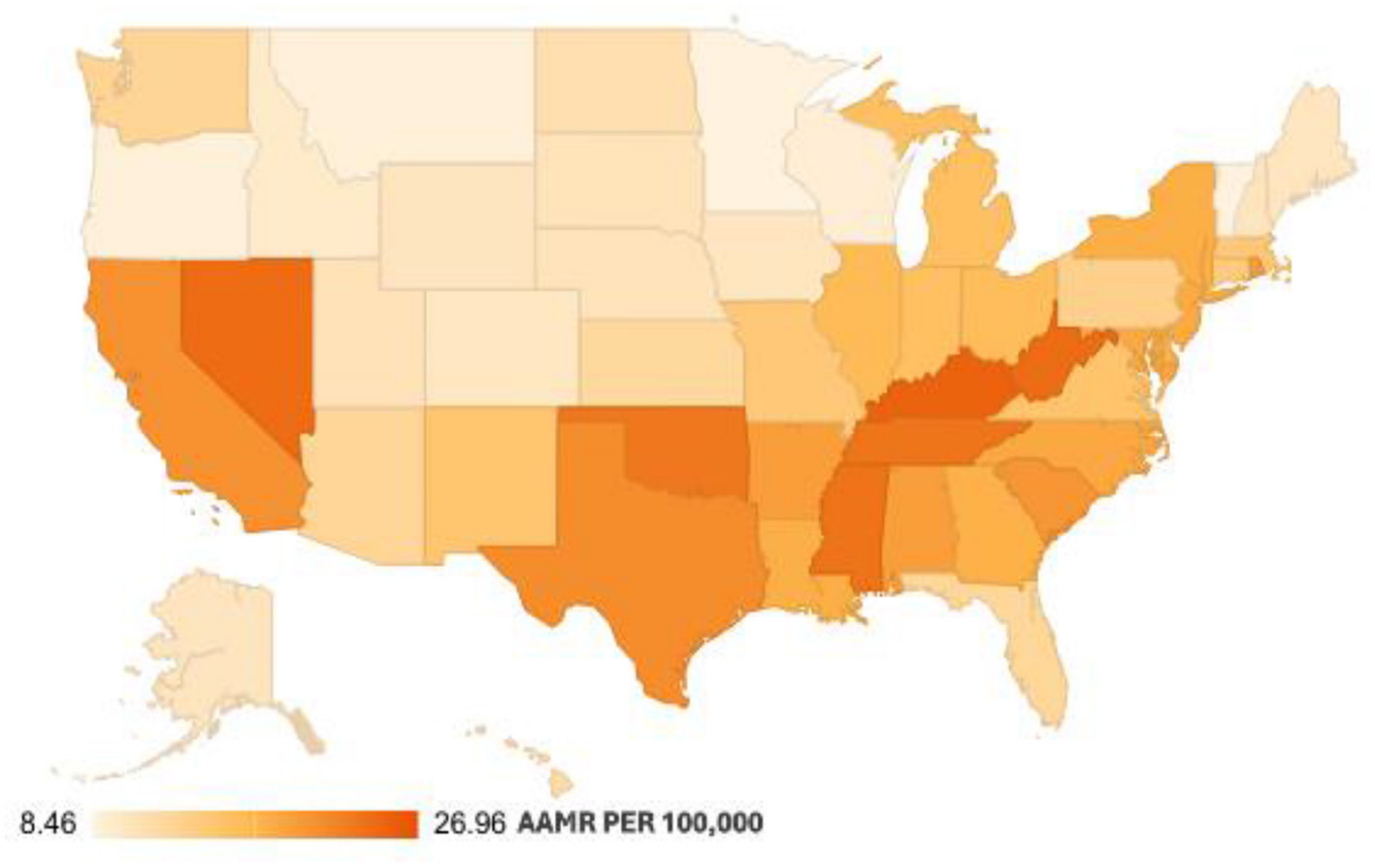
Pneumonia and sepsis-related age-adjusted mortality rates per 100,000, stratified by states among adults (Aged ≥25) in the United States, 1999 to 2020.

Mortality trends showed significant disparities across the different states, with Vermont having the lowest AAMR of 8.46 (95% CI: 7.90 to 9.01) and the District of Columbia having the highest AAMR of 26.96 (95% CI: 25.85 to 28.08) (Figure 3, Supplementary Table 6). States that fell into the top 90th percentile were the District of Columbia, Kentucky, Nevada, West Virginia, and Tennessee, compared with states that fell into the lower 10th percentile, including Vermont, Minnesota, Montana, Oregon, and Wisconsin.

Over the course of the study, the South and Midwest regions experienced an incline from 1999 to 2018 with an APC of 0.78 (95% CI: 0.22 to 1.34) (*p-*value: 0.009) and 0.91 (95% CI: 0.38 to 1.45) (*p-*value: 0.002), respectively, following a significant surge in both from 2018 to 2020 with an APC of 19.50 (95% CI: 3.29 to 38.25) (*p-*value: 0.019) and 18.35 (95% CI: 2.10 to 37.19) (*p-*value: 0.028), respectively. While the western region had a steady increase with an APC of 1.27 (95% CI: 0.67 to 1.87) (*p-*value: 0.0002). The only decline was observed in the Northeast region from 1999 to 2011 with an APC of −1.28 (95% CI: −2.47 to −0.07) (*p-*value: 0.039), followed by a surge from 2011 to 2020 with an APC of 2.73 (95% CI: 1.04 to 4.45) (*p-*value: 0.003). The highest mortality was observed in the Southern region (AAMR: 18.82; 95% CI: 18.75 to 18.88), followed by the Western (AAMR: 16.92; 95% CI: 16.84 to 17), Northeastern (AAMR: 16.21; 95% CI: 16.12 to 16.29), and Midwestern regions (AAMR: 14.45; 95% CI: 14.38 to 14.53) (Supplementary Tables 3, 7).

**Figure 4:**
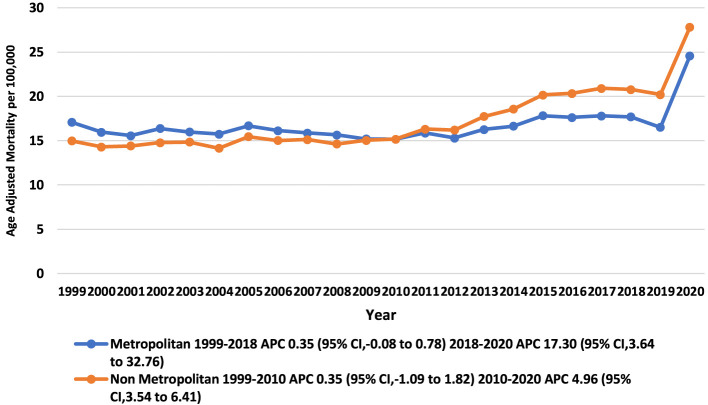
Trends in pneumonia and sepsis-related age-adjusted mortality rates per 100,000, stratified by urbanization among adults (Aged ≥25) in the United States, 1999 to 2020.

From 1999 to 2020, the pneumonia- and sepsis-related AAMRs were marginally higher in non-metropolitan areas (overall AAMR: 17.32, 95% CI: 17.23 to 17.41) as compared to metropolitan areas (overall AAMR: 16.86, 95% CI: 16.82 to 16.9). The metropolitan trend had an initial period of gradual incline followed by a sudden significant surge from 2018 to 2020 with an APC of 17.30 (95% CI: 3.6381 to 32.7653) (*p-*value: 0.015), while the non-metropolitan trend surged from 2010 to 2020 with an APC of 4.96 (95% CI: 3.5389 to 6.4099) (*p-*value: 0.000001) (Figure 4, Supplementary Tables 3, 8).

## 4 Discussion

This 21-year analysis of mortality data from the Centers for Disease Control and Prevention indicates several important findings. First, while the number of deaths attributed to pneumonia and sepsis generally trended upwards, with a notable surge in 2020 coinciding with the COVID-19 pandemic, trends varied by demographic subgroup. Second, males showed higher AAMRs than females, with adults particularly over 65 years showing a higher death rate compared to younger adults. Third, Non-Hispanic African Americans had the highest AAMR amongst the other racial groups. States such as the District of Columbia, Kentucky, Mississippi, Nevada, Oklahoma, South Carolina, Tennessee, Texas, and West Virginia recorded significantly higher AAMRs than the rest of the states. In addition, a significant contrast was observed between metropolitan and non-metropolitan settings, with non-metropolitan areas settings exhibiting higher AAMRs than their metropolitan counterparts. These findings highlight the significant consequences of sepsis and pneumonia while also revealing crucial disparities that influence vulnerable populations and specific regions.

Our findings highlight the strong interplay between pneumonia and sepsis in infection-related mortality. Pneumonia is one of the most common precipitating infections leading to sepsis and septic shock, and in many fatal cases, both conditions are documented on death certificates. By restricting inclusion to deaths where both pneumonia and sepsis were recorded, we aimed to capture severe infection-associated mortality reflecting systemic progression rather than isolated localized infection or non-pulmonary sepsis. While prior studies have examined pneumonia or sepsis independently (6), our approach provides a clinically meaningful estimate of systemic infection mortality within population-level data.

These results are consistent with previous studies documenting the burden of respiratory and septic illnesses in the US. Many studies have highlighted sepsis as a leading contributor to the global health burden (6). Although hospital case-fatality rates have decreased, national analyses continue to report increasing sepsis-related mortality in the United States. Rising sepsis mortality figures warrant a conservative interpretation due to the potential influence of ascertainment bias. Recent shifts toward Sepsis-3 definitions and enhanced financial incentives for quality reporting align with more aggressive documentation on death records. These factors often create an artificial inflation of mortality rates that exists independently of true disease prevalence (25, 26). Similarly, pneumonia has remained the leading cause of infectious mortality despite advances in vaccination and antimicrobial therapy (27). Our methodology which used national mortality data from the CDC WONDER database, corresponds to prior studies on sepsis. A prior study demonstrated persistent sex, race, and geographic disparities, with sepsis mortality showing a robust increase between 2019–2021 due to the COVID-19 pandemic (28). While our data mirrors this spike, it is critical to acknowledge the confounding role of COVID-19. We did not formally separate COVID-related deaths, and given that viral pneumonia and sepsis are primary pathways of death in severe COVID-19, the 2020 surge likely represents a composite of pre-existing sepsis trends and misattributed or co-occurring COVID-19 mortality. Documentation practices during the pandemic fluctuated, where sepsis occasionally went unrecorded as an underlying cause in favor of COVID-19 (45). In other instances, deaths lacking definitive test results appeared as pneumonia or sepsis rather than COVID-19 on official certificates (45). This variability suggests that certification trends track more with diagnostic availability than with actual disease incidence (45). Our findings are also consistent with these patterns, showing higher AAMR in men, older adults, and residents of non-metropolitan areas, with a sudden spike in 2020. These methodological techniques are also reflected in our use of CDC WONDER database, which provides insightful comparisons to support the validity of our findings and trends.

Biological frameworks highlight that males frequently demonstrate more intense proinflammatory activity during infectious episodes. Conversely, estrogen levels in females correlate with specific immune-modulating effects that appear to provide a protective advantage against such insults. These variations correspond with existing literature on systemic biological and sociological responses to infectious insults (29). Sex-specific differences in pathogen response appear linked to behavioral variables like tobacco or alcohol use alongside higher comorbidity rates in men (29–32). Conversely, data suggest that women frequently engage with preventive services and seek clinical care at earlier stages of illness (33). Older age groups showed higher mortality, a trend that tracks with natural immunosenescence and diminished physiological reserves (34, 35). Racial disparities affecting African Americans reflect a complex interplay of systemic inequities, reduced health care access, and socioeconomic disadvantage, in addition to higher prevalence of comorbidities like diabetes, hypertension and chronic kidney disease (36–38). Within African American communities, the 2020 mortality spike mirrors historical disparities in respiratory and systemic infection trends (39, 40). Similarly, non-metropolitan areas showed higher death counts compared to urban centers. These regional differences frequently occur alongside limited healthcare infrastructure, lower community health literacy, and reduced vaccination coverage among aging rural residents (41–43).

The clinical and policy implications of these findings are of paramount importance. Future health policies should be tailored, focusing on high-risk groups and the potential factors responsible for the current mortality trends to ensure more targeted prevention and intervention strategies in order to mitigate mortality trends. Enhanced early detection measures for sepsis, such as the broad use of sepsis bundles, electronic alert systems, and improved triage protocols, may reduce mortality (44) and should be implemented fairly across hospital systems. Public health initiatives should prioritize expanded coverage for pneumococcal, influenza, and booster vaccinations among geriatric populations and high-risk racial cohorts. Enhancing healthcare reach in non-metropolitan regions involves implementing telemedicine platforms, deploying mobile critical care units, and offering structural incentives for physician placement in rural areas. Furthermore, emerging diagnostic and monitoring approaches, including hyperpolarized gas magnetic resonance imaging for rapid assessment of lung ventilation, may support earlier characterization and follow-up of pulmonary disease in selected settings (46, 47). In parallel, detailed CT-based characterization of pneumonitis phenotypes may aid clinical differentiation in complex respiratory presentations (48). Although such tools were not evaluated in the present CDC-based mortality analysis, they represent evolving diagnostic advances that may be relevant when interpreting future population-level mortality patterns.

Validation of these ecological observations requires future investigations that utilize granular, individual-level clinical records. Ongoing surveillance of mortality trends in the years following 2020 serves to clarify whether the recorded surge represents a transient spike or a fundamental epidemiologic transition. Rigorous assessment of the associations between sex, race, and immunological profiles remains necessary to clarify the biological patterns linked to such disparities. Efforts should be made toward integrating advanced surveillance systems into healthcare to effectively evaluate mortality rates, health status, hospital admissions, and socioeconomic data.

## 5 Study limitations

There are several limitations that need to be considered in this study. First, the dependence on death certificates and ICD Codes can potentially under- or overestimate the mortality trends. Specifically, changes in coding practices over the 21-year period, such as the transition from ICD-9 to ICD-10 and the widespread adoption of Sepsis-3 definitions, may introduce temporal bias that distinguishes documentation trends from true disease burden. Second, as an ecological study based on aggregate data, we cannot make causal inferences regarding individual-level mechanisms (e.g., immune response or healthcare-seeking behavior); our explanations regarding these factors remain hypotheses based on existing literature. Third, this study does not provide information on clinical variables like vitals, laboratory findings, radiographic findings, microbiological findings, thereby limiting pathogen-specific analysis, as well as biomarkers of disease severity that could have provided a more nuanced understanding of pneumonia and sepsis. Additionally, evaluation of diagnostic strategies and their impact on mortality was beyond the scope of this population-level analysis. Fourth, the surge in mortality observed in 2020 is heavily confounded by the COVID-19 pandemic. We were unable to isolate deaths where COVID-19 was a coinfection vs. the primary cause, limiting our ability to interpret the 2018–2020 trend as a continuity of pre-pandemic sepsis epidemiology. Additionally, important factors and confounders such as vaccination status, detailed patient-level comorbidities (e.g., chronic diseases, malignancies, and metabolic disorders), and treatment differences were not available. Moreover, the cause of death from out-of-hospital reported deaths is known to be less reliable. Furthermore, examining pneumonia and sepsis jointly may limit direct comparisons with studies focusing on a single diagnosis. Future sensitivity analyses exploring pneumonia-only vs. sepsis-only mortality could provide complementary insights and help clarify how these conditions interact in shaping population-level trends. Finally, although these datasets provide large-scale insights, they may obscure important local or regional trends.

## 6 Conclusion

Mortality attributed to pneumonia and sepsis has shown an upward trend from 1999 to 2020, with a sharp escalation during the COVID-19 pandemic. While this rise may partly reflect evolving coding practices and the confounding impact of COVID-19, the data indicate that mortality disproportionately affected men, older adults, NH African Americans, and residents of non-metropolitan areas. The biological and clinical link between pneumonia and sepsis shows the importance of analyzing them together. Thus, it is crucial to address these disparities, which requires coordinated efforts across clinical, public health, and policy sectors to improve both disease management and the accuracy of cause-of-death reporting.

## Data Availability

The original contributions presented in the study are included in the article/Supplementary material, further inquiries can be directed to the corresponding author.
