# Supplementary material for: Demographic and regional trends in pneumonia and sepsis related mortality in the U.S., 1999–2020: a CDC wonder database analysis
Source: Front Public Health. 2026 May 13;14:1715043. doi: 10.3389/fpubh.2026.1715043 (PMC13212507; doi:10.3389/fpubh.2026.1715043)

**SUPPLEMENTARY APPENDIX**

## **Supplementary Table 1:** Absolute number of Pneumonia and Sepsis-related deaths per 100,000 among individuals aged 25-85+ years stratified by sex and race in the United States, 1999-2020

NH- Non Hispanic

| **Year** | **Overall** | **Male** | **Female** | **NH Black or African American** | **NH-American Indian or Alaska Native** | **NH-Asian or Pacific Islander** | **NH White** | **Hispanic or**  **Latino** |
| --- | --- | --- | --- | --- | --- | --- | --- | --- |
| 1999 | 29368 | 14738 | 14630 | 4712 | 147 | 620 | 22076 | 1696 |
| 2000 | 27953 | 14180 | 13773 | 4363 | 140 | 617 | 21170 | 1554 |
| 2001 | 27864 | 14091 | 13773 | 4283 | 136 | 655 | 20950 | 1735 |
| 2002 | 29593 | 15021 | 14572 | 4417 | 146 | 688 | 22327 | 1910 |
| 2003 | 29597 | 15041 | 14556 | 4362 | 191 | 706 | 22312 | 1935 |
| 2004 | 29388 | 14975 | 14413 | 4312 | 161 | 769 | 22062 | 1994 |
| 2005 | 31936 | 16114 | 15822 | 4476 | 209 | 855 | 23986 | 2336 |
| 2006 | 31537 | 16218 | 15319 | 4409 | 185 | 956 | 23631 | 2290 |
| 2007 | 31802 | 16452 | 15350 | 4583 | 207 | 950 | 23603 | 2402 |
| 2008 | 31889 | 16083 | 15806 | 4648 | 244 | 1032 | 23419 | 2479 |
| 2009 | 31854 | 16383 | 15471 | 4539 | 267 | 1021 | 23299 | 2675 |
| 2010 | 32416 | 16756 | 15660 | 4512 | 248 | 1118 | 23747 | 2715 |
| 2011 | 35026 | 17996 | 17030 | 4800 | 310 | 1184 | 25740 | 2899 |
| 2012 | 34816 | 17815 | 17001 | 4846 | 296 | 1227 | 25349 | 2976 |
| 2013 | 38066 | 19628 | 18438 | 5317 | 314 | 1384 | 27640 | 3279 |
| 2014 | 40018 | 20655 | 19363 | 5413 | 355 | 1415 | 28989 | 3646 |
| 2015 | 43937 | 22696 | 21241 | 5953 | 387 | 1606 | 31947 | 3814 |
| 2016 | 44489 | 23173 | 21316 | 5937 | 370 | 1642 | 32408 | 3965 |
| 2017 | 46115 | 23954 | 22161 | 6122 | 421 | 1739 | 33393 | 4249 |
| 2018 | 46873 | 24712 | 22161 | 6425 | 382 | 1774 | 33744 | 4386 |
| 2019 | 44952 | 23736 | 21216 | 6193 | 403 | 1781 | 32299 | 4158 |
| 2020 | 66945 | 37533 | 29412 | 10681 | 718 | 2856 | 42272 | 10238 |
| Total | 806434 | 417950 | 388484 | 115303 | 6237 | 26595 | 586363 | 69331 |

**Supplementary Table 2:** Absolute number of Pneumonia and Sepsis-related deaths among individuals aged 25-85+ years stratified by place of death in the United States, 1999-2020

| **Year** | **Medical Facility - Inpatient** | **Medical Facility - Dead on Arrival** | **Medical Facility - Status unknown** | **Medical Facility -Outpatient** | **Decedent’s Home** | **Hospice Facility** | **Nursing Home/Long Term Care Facility** | **Other** | **Place of Death Unknown** |
| --- | --- | --- | --- | --- | --- | --- | --- | --- | --- |
| 1999 | 24725 | 43 | 205 | 824 | 450 | Unknown | 2965 | 150 | Unknown |
| 2000 | 23602 | 44 | 219 | 785 | 389 | Unknown | 2758 | 149 | Unknown |
| 2001 | 23785 | 42 | 181 | 797 | 381 | Unknown | 2521 | 152 | Unknown |
| 2002 | 25394 | 39 | 216 | 862 | 362 | Unknown | 2512 | 199 | Unknown |
| 2003 | 25379 | 33 | Unknown | 904 | 430 | 12 | 2470 | 234 | 135 |
| 2004 | 25252 | 28 | Unknown | 881 | 458 | 36 | 2315 | 290 | 128 |
| 2005 | 27256 | 27 | Unknown | 973 | 586 | 145 | 2501 | 284 | 164 |
| 2006 | 27366 | 22 | Unknown | 964 | 515 | 199 | 2134 | 242 | 95 |
| 2007 | 27716 | 26 | Unknown | 970 | 484 | 308 | 2001 | 250 | 47 |
| 2008 | 27383 | 28 | Unknown | 951 | 469 | 374 | 1907 | 241 | 536 |
| 2009 | 27024 | 17 | Unknown | 945 | 492 | 462 | 1786 | 262 | 866 |
| 2010 | 28146 | 23 | Unknown | 958 | 525 | 601 | 1812 | 314 | 37 |
| 2011 | 30339 | 19 | Unknown | 1033 | 629 | 746 | 1853 | 361 | 46 |
| 2012 | 29958 | 21 | Unknown | 1045 | 632 | 921 | 1855 | 361 | 23 |
| 2013 | 32740 | 50 | Unknown | 1132 | 741 | 1077 | 1862 | 438 | 26 |
| 2014 | 34342 | 17 | Unknown | 1191 | 732 | 1409 | 1978 | 325 | 24 |
| 2015 | 37038 | 23 | Unknown | 1341 | 936 | 2029 | 2254 | 297 | 19 |
| 2016 | 37482 | 18 | Unknown | 1336 | 1088 | 2088 | 2130 | 342 | Unknown |
| 2017 | 38353 | 24 | Unknown | 1482 | 1187 | 2519 | 2207 | 336 | Unknown |
| 2018 | 38914 | 19 | Unknown | 1488 | 1298 | 2555 | 2256 | 335 | Unknown |
| 2019 | 37314 | 13 | Unknown | 1513 | 1270 | 2469 | 1979 | 389 | Unknown |
| 2020 | 57318 | 21 | Unknown | 2161 | 1947 | 2726 | 2238 | 523 | 11 |
| Total | 686826 | 597 | 821 | 24536 | 16001 | 20676 | 48294 | 6474 | 2209 |

**Supplementary Table 3:** APC (Annual Percent Change) of Pneumonia and Sepsis-related AAMR per 100,000 in the United States from 1999 to 2020

| **Year Interval** | **APC (95% CI)** |
| --- | --- |
| **Overall** | |
| 1999-2018 | 0.61* (0.15 to 1.07) |
| 2018-2020 | 17.15* (3.63 to 32.43) |
| **Male** | |
| 1999-2018 | 0.27 (-0.20 to 0.74) |
| 2018-2020 | 20.24* (6.17 to 36.16) |
| **Female** | |
| 1999-2020 | 1.34* (0.76 to 1.93) |
| **NH Black or African American** | |
| 1999-2018 | -1.04* (-1.43 to -0.64) |
| 2018-2020 | 25.96* (13.02 to 40.38) |
| **NH-American Indian or Alaska Native** | |
| 1999-2020 | 2.93* (1.86 to 3.99) |
| **NH-Asian or Pacific Islander** | |
| 1999-2018 | -0.58* (-0.86 to -0.29) |
| 2018-2020 | 16.08 (8.93 to 23.70) |
| **NH White** | |
| 1999-2010 | -0.33 (-1.49 to 0.84) |
| 2010-2020 | 3.13* (1.94 to 4.34) |
| **Hispanic or Latino** | |
| 1999-2018 | -0.52* (-0.84 to -0.19) |
| 2018-2020 | 41.37* (31.43 to 52.06) |
| **Metropolitan** | |
| 1999-2018 | 0.35 (-0.08 to 0.78) |
| 2018-2020 | 17.30* (3.64 to 32.76) |
| **Non-Metropolitan** | |
| 1999-2010 | 0.35 (-1.09 to 1.82) |
| 2010-2020 | 4.96* (3.54 to 6.41) |
| **Midwest** | |
| 1999-2018 | 0.91* (0.38 to 1.45) |
| 2018-2020 | 18.35* (2.09 to 37.19) |
| **Northeast** | |
| 1999-2011 | -1.28* (-2.47 to -0.07) |
| 2011-2020 | 2.73* (1.04 to 4.45) |
| **South** | |
| 1999-2018 | 0.78* (0.22 to 1.34) |
| 2018-2020 | 19.50* (3.28 to 38.25) |
| **West** | |
| 1999-2020 | 1.27* (0.67 to 1.87) |

## **Supplementary Table 4:** Pneumonia and Sepsis-related Age-Adjusted Mortality Rates per 100,000 among patients aged 25-85+ years stratified by sex in the United States, 1999-2020

| **Year** | **Overall** | **Male** | **Female** |
| --- | --- | --- | --- |
| 1999 | 16.66 | 21.5 | 13.57 |
| 2000 | 15.66 | 20.44 | 12.63 |
| 2001 | 15.35 | 19.83 | 12.52 |
| 2002 | 16.05 | 20.75 | 13.07 |
| 2003 | 15.81 | 20.2 | 12.87 |
| 2004 | 15.43 | 19.62 | 12.64 |
| 2005 | 16.45 | 20.64 | 13.65 |
| 2006 | 15.92 | 20.2 | 13.04 |
| 2007 | 15.74 | 20.01 | 12.9 |
| 2008 | 15.46 | 19.02 | 13 |
| 2009 | 15.15 | 18.88 | 12.61 |
| 2010 | 15.16 | 19.04 | 12.5 |
| 2011 | 15.92 | 19.67 | 13.28 |
| 2012 | 15.41 | 18.89 | 12.97 |
| 2013 | 16.49 | 20.18 | 13.82 |
| 2014 | 16.94 | 20.61 | 14.31 |
| 2015 | 18.18 | 22.14 | 15.29 |
| 2016 | 18.06 | 21.99 | 15.14 |
| 2017 | 18.28 | 22.19 | 15.44 |
| 2018 | 18.18 | 22.25 | 15.11 |
| 2019 | 17.08 | 20.89 | 14.24 |
| 2020 | 25.06 | 31.91 | 19.67 |
| Total | 16.92 | 21.16 | 13.94 |

## **Supplementary Table 5:** Pneumonia and Sepsis-related Age-Adjusted Mortality Rates per 100,000 among patients aged 25-85+ years stratified by race in the United States, 1999-2020

| **Year** | **NH Black or African American** | **NH-American Indian or Alaska Native** | **NH-Asian or Pacific Islander** | **NH White** | **Hispanic or Latino** |
| --- | --- | --- | --- | --- | --- |
| 1999 | 31.36 | 20.46 | 16.54 | 14.84 | 20.11 |
| 2000 | 29.16 | 17.58 | 15.42 | 14.11 | 17.58 |
| 2001 | 27.97 | 16.46 | 15.76 | 13.8 | 18.46 |
| 2002 | 28.69 | 16.87 | 15.3 | 14.57 | 19.54 |
| 2003 | 27.66 | 20.75 | 14.56 | 14.38 | 18.49 |
| 2004 | 26.93 | 17.8 | 15.21 | 14.05 | 18.25 |
| 2005 | 27.22 | 22.81 | 15.46 | 15.09 | 20.11 |
| 2006 | 26 | 18.96 | 16.69 | 14.65 | 18.58 |
| 2007 | 26.22 | 20.12 | 15.46 | 14.4 | 18.49 |
| 2008 | 25.97 | 23.78 | 15.79 | 14.09 | 18.03 |
| 2009 | 24.41 | 24.49 | 14.98 | 13.84 | 18.08 |
| 2010 | 23.8 | 21.75 | 15.42 | 13.9 | 18.07 |
| 2011 | 24.5 | 26.11 | 15.24 | 14.75 | 17.85 |
| 2012 | 23.71 | 24.85 | 14.87 | 14.24 | 17.19 |
| 2013 | 25.1 | 24.31 | 15.3 | 15.31 | 17.96 |
| 2014 | 24.71 | 26.49 | 14.54 | 15.8 | 18.67 |
| 2015 | 26.25 | 27.44 | 15.57 | 17.14 | 18.58 |
| 2016 | 25.35 | 25.17 | 14.87 | 17.2 | 18.21 |
| 2017 | 25.22 | 27.8 | 14.88 | 17.44 | 18.81 |
| 2018 | 25.72 | 24.12 | 14.4 | 17.3 | 18.41 |
| 2019 | 24.1 | 24.18 | 13.77 | 16.3 | 16.68 |
| 2020 | 40.06 | 40.47 | 20.69 | 21.22 | 37.62 |
| Total | 26.84 | 23.96 | 15.48 | 15.51 | 19.63 |

NH- Non Hispanic

## **Supplementary Table 6:** Pneumonia and Sepsis-related Age-Adjusted Mortality Rates per 100,000 among patients aged 25-85+ years stratified by state in the United States, 1999-2020

| **State** | **Deaths** | **Age-Adjusted Mortality Rate** |
| --- | --- | --- |
| Alabama | 8183 | 19.95 |
| Alaska | 724 | 10.55 |
| Arizona | 8602 | 12.99 |
| Arkansas | 5432 | 19.98 |
| California | 78950 | 21.1 |
| Colorado | 8208 | 10.36 |
| Connecticut | 6273 | 14.7 |
| Delaware | 2128 | 17.92 |
| District of Columbia | 947 | 26.96 |
| Florida | 32591 | 12.81 |
| Georgia | 10292 | 18.22 |
| Hawaii | 3104 | 11.98 |
| Idaho | 3511 | 9.84 |
| Illinois | 21107 | 17.05 |
| Indiana | 16257 | 16.3 |
| Iowa | 8429 | 10.47 |
| Kansas | 5582 | 12.64 |
| Kentucky | 11279 | 25.29 |
| Louisiana | 7165 | 18.43 |
| Maine | 3265 | 10.42 |
| Maryland | 12953 | 19.02 |
| Massachusetts | 11755 | 15.03 |
| Michigan | 20220 | 16.33 |
| Minnesota | 14544 | 8.59 |
| Mississippi | 5574 | 23.9 |
| Missouri | 12899 | 15.03 |
| Montana | 2131 | 8.86 |
| Nebraska | 4936 | 10.76 |
| Nevada | 2255 | 24.51 |
| New Hampshire | 2965 | 10.66 |
| New Jersey | 17362 | 18.81 |
| New Mexico | 2726 | 15.54 |
| New York | 29855 | 18.4 |
| North Carolina | 22077 | 19.03 |
| North Dakota | 1990 | 11.5 |
| Ohio | 38712 | 16.75 |
| Oklahoma | 10252 | 23.47 |
| Oregon | 11699 | 8.88 |
| Pennsylvania | 36378 | 13.79 |
| Rhode Island | 3175 | 19.8 |
| South Carolina | 12220 | 20.66 |
| South Dakota | 2162 | 10.9 |
| Tennessee | 16124 | 23.72 |
| Texas | 47834 | 21.54 |
| Utah | 3091 | 11.07 |
| Vermont | 2366 | 8.46 |
| Virginia | 12901 | 15.76 |
| Washington | 17646 | 13.08 |
| West Virginia | 6442 | 24.46 |
| Wisconsin | 15206 | 9.24 |
| Wyoming | 1118 | 10.56 |
| Total | 543,597 | 16.92 |

## **Supplementary Table 7:** Pneumonia and Sepsis-related Age-Adjusted Mortality Rates per 100,000 among patients aged 25-85+ years stratified by census region in the United States, 1999-2020

| **Year** | **Northeast** | **Midwest** | **South** | **West** |
| --- | --- | --- | --- | --- |
| 1999 | 11.47 | 12.94 | 10.91 | 10.94 |
| 2000 | 11.08 | 12.66 | 10.67 | 10.29 |
| 2001 | 11.77 | 12.98 | 11.14 | 11.55 |
| 2002 | 11.47 | 13.35 | 11.46 | 11.96 |
| 2003 | 10.89 | 13.48 | 11.5 | 11.76 |
| 2004 | 10.93 | 13.21 | 11.14 | 11.88 |
| 2005 | 11.43 | 13.98 | 11.72 | 12.36 |
| 2006 | 10.47 | 13.4 | 11.41 | 12.62 |
| 2007 | 10.63 | 13.77 | 11.48 | 12.41 |
| 2008 | 10.78 | 14 | 11.85 | 12.69 |
| 2009 | 10.3 | 13.9 | 11.46 | 12.06 |
| 2010 | 11.1 | 13.82 | 11.7 | 12.9 |
| 2011 | 11.79 | 14.47 | 11.86 | 14.04 |
| 2012 | 11.81 | 14.5 | 12.42 | 13.89 |
| 2013 | 11.97 | 14.38 | 12.97 | 14.73 |
| 2014 | 12.17 | 15.03 | 12.68 | 14.33 |
| 2015 | 12.44 | 16.06 | 13.37 | 15.4 |
| 2016 | 12.43 | 15.68 | 13.83 | 15.91 |
| 2017 | 12.64 | 16.97 | 14.84 | 17.04 |
| 2018 | 12.92 | 17.66 | 15.2 | 16.98 |
| 2019 | 13.34 | 18.1 | 16.11 | 17.21 |
| 2020 | 17.68 | 22.31 | 20.39 | 21.04 |
| Total | 11.97 | 15.01 | 13.01 | 14.21 |

## **Supplementary Table 8:** Pneumonia and Sepsis-related Age-Adjusted Mortality Rates per 100,000 among patients aged 25-85+ years stratified by urban-rural classification in the United States, 1999-2020

| **Year** | **Metropolitan** | **Non-metropolitan** |
| --- | --- | --- |
| 1999 | 17.07 | 14.97 |
| 2000 | 15.97 | 14.3 |
| 2001 | 15.57 | 14.41 |
| 2002 | 16.37 | 14.78 |
| 2003 | 15.99 | 14.85 |
| 2004 | 15.73 | 14.14 |
| 2005 | 16.69 | 15.45 |
| 2006 | 16.14 | 15.01 |
| 2007 | 15.88 | 15.13 |
| 2008 | 15.66 | 14.63 |
| 2009 | 15.19 | 15.04 |
| 2010 | 15.17 | 15.17 |
| 2011 | 15.87 | 16.3 |
| 2012 | 15.3 | 16.2 |
| 2013 | 16.26 | 17.73 |
| 2014 | 16.64 | 18.56 |
| 2015 | 17.83 | 20.17 |
| 2016 | 17.62 | 20.33 |
| 2017 | 17.79 | 20.9 |
| 2018 | 17.68 | 20.77 |
| 2019 | 16.51 | 20.2 |
| 2020 | 24.56 | 27.79 |
| Total | 16.86 | 17.32 |

**Supplementary Table 9:** Pneumonia and Sepsis-related Age-Adjusted Mortality Rates per 100,000 stratified by age group in the United States from 1999 to 2020

| **Year** | **25-44** | **45-64** | **65-85+** |
| --- | --- | --- | --- |
| 1999 | 1.56 | 7.17 | 68.97 |
| 2000 | 1.2 | 6.75 | 65.41 |
| 2001 | 1.3 | 6.61 | 63.85 |
| 2002 | 1.25 | 7.05 | 66.77 |
| 2003 | 1.3 | 7.14 | 65.27 |
| 2004 | 1.15 | 7.11 | 63.72 |
| 2005 | 1.25 | 7.57 | 67.91 |
| 2006 | 1.25 | 7.47 | 65.36 |
| 2007 | 1.2 | 7.59 | 64.37 |
| 2008 | 1.35 | 7.69 | 62.4 |
| 2009 | 1.55 | 7.74 | 60.27 |
| 2010 | 1.3 | 7.45 | 61.4 |
| 2011 | 1.39 | 8.17 | 63.82 |
| 2012 | 1.29 | 8.11 | 61.53 |
| 2013 | 1.45 | 8.68 | 65.73 |
| 2014 | 1.45 | 9.24 | 67.01 |
| 2015 | 1.51 | 9.3 | 73.11 |
| 2016 | 1.66 | 9.75 | 71.33 |
| 2017 | 1.61 | 9.57 | 72.93 |
| 2018 | 1.61 | 9.99 | 71.67 |
| 2019 | 1.61 | 9.25 | 67.33 |
| 2020 | 2.65 | 16.01 | 93.81 |
| Total | 1.45 | 8.58 | 68.08 |

**Supplementary Figure 1.** Yearly trends in Pneumonia and Sepsis-related AAMR per 100,000 in the United States from 1999 to 2020

**Supplementary Figure 2.** Trends in Pneumonia and Sepsis-related AAMR per 100,000 stratified by sex in the United States from 1999 to 2020

**Supplementary Figure 3.** Trends in Pneumonia and Sepsis-related AAMR per 100,000 stratified by ten year age-groups in the United States from 1999 to 2020

**Supplementary Figure 4.** Pneumonia and Sepsis-related AAMR per 100,000 stratified by census region in the United States from 1999 to 2020


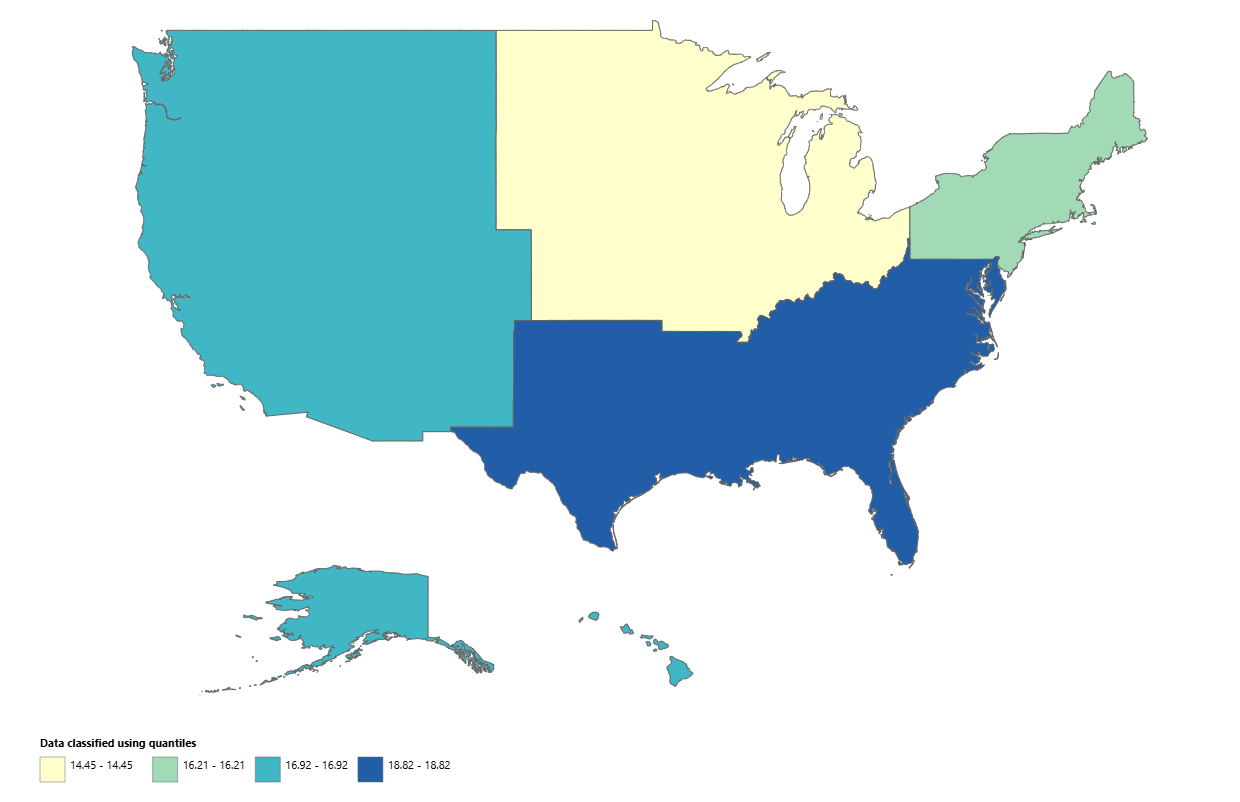

Supplement: Supplementary file 1 [file Data_Sheet_1.docx]
